# Metagenomic Study on Chinese Homemade Paocai: The Effects of Raw Materials and Fermentation Periods on the Microbial Ecology and Volatile Components

**DOI:** 10.3390/foods11010062

**Published:** 2021-12-28

**Authors:** Linjun Jiang, Shuang Xian, Xingyan Liu, Guanghui Shen, Zhiqing Zhang, Xiaoyan Hou, Anjun Chen

**Affiliations:** College of Food Science, Sichuan Agricultural University, Ya’an 625014, China; jianglinjun2020@163.com (L.J.); xianshuang@stu.sicau.edu.cn (S.X.); lxy05@126.com (X.L.); Shenghuishen@163.com (G.S.); zqzhang721@163.com (Z.Z.); houxiaoyan106@163.com (X.H.)

**Keywords:** red radish, cabbage, fermented foods, microbial ecology, flavor components

## Abstract

“Chinese paocai” is typically made by fermenting red radish or cabbage with aged brine (6–8 *w*/*w*). This study aimed to reveal the effects of paocai raw materials on fermentation microorganisms by metagenomics sequencing technology, and on volatile organic compounds (VOCs) by gas chromatography–mass spectroscopy, using red radish or cabbage fermented for six rounds with aged brine. The results showed that in the same fermentation period, the microbial diversity in cabbage was higher than that in red radish. *Secundilactobacillus paracollinoides* and *Furfurilactobacillus siliginis* were the characteristic bacteria in red radish paocai, whereas 15 species of characteristic microbes were found in cabbage. Thirteen kinds of VOCs were different between the two raw materials and the correlation between the microorganisms and VOCs showed that cabbage paocai had stronger correlations than radish paocai for the most significant relationship between 4-isopropylbenzyl alcohol, α-cadinol, terpinolene and isobutyl phenylacetate. The results of this study provide a theoretical basis for understanding the microbiota and their relation to the characteristic flavors of the fermented paocai.

## 1. Introduction

Fermentation is a widely used technology to preserve foods, improve nutritional value and extend shelf-life, and fermented vegetables are popular and traditional in Asian countries [1]. Among the fermented vegetables, “paocai” is a beloved food with a history of more than 3000 years, which are typically consumed as side dishes or appetizers and are characterized by their tender, crisp texture and rich health benefits owing to the lactic-acid bacteria (LAB) [2,3,4]. Vegetables for making paocai are usually immersed in 6–8% (*w*/*w*) sodium chloride solution and allowed to undergo spontaneous anaerobic or microaerobic fermentation by the epiphytic microbes (mostly LAB) present on the raw materials for 6–10 days [5].

The microbial composition and flavor in paocai fermentation systems are related to various factors, especially the raw materials. Previous studies have found that the microbial niches in raw ingredients determine the eventual microbial community [6], this conclusion has been confirmed in some studies on microorganisms in Sichuan paocai; for instance, *Rosenbergiella*, *Staphylococcus*, *Hyphopichia* and *Kodamaea* dominated the fermentation of chili pepper [7], while *Lactobacillus*, *Leuconostoc*, *Achromobacter* and *Pediococcus* were the main microbial organisms during the fermentation of cabbage pickle [8]. This is due to the fact that the spontaneous fermentation of vegetable products is highly dependent on the naturally occurring LAB present on the raw materials. A large amount of different LAB can be accumulated in the aged brine in the process of continuous periodic fermentation, and the total viable count can be up to 8.79 Log CFU/mL [9]. It is worth noting that the microorganisms were shown to rapidly build a stable fermentation system at the initial stages and finally obtain paocai products with different flavors. Due to the complex microbial ecology in the fermentation system, two or three strains of artificially isolated LAB are often used in industry to accelerate the fermentation process and to achieve a uniform quality of the products [10]. However, based on consumer surveys, the quality of the final products is not as good as homemade paocai. Homemade paocai usually uses aged brine as the starter, which is brine fermented for many years and even decades [11,12]. The microorganisms in the fermentation system metabolize the nutrients in the raw materials to produce acids, that makes the fermented vegetables have a unique flavor [13], which is another source of paocai flavor besides that of the raw materials. Therefore, understanding the effects of raw materials and fermentation periods on the microbial ecology and volatile components is of far-reaching significance for understanding how microorganisms construct stable fermentation systems.

Metagenomics sequencing technology can extract all microbial DNA from the fermentation system, construct a metagenomic PE library and use genomics research strategy to study the genetic composition and gene function of all microorganisms contained in environmental samples. This technique can avoid amplification and sequencing bias, which commonly occurred in other sequencing techniques [14]. Metagenomics, although still underexploited, can reveal the succession and function of microorganisms and establish the relationship between microorganisms and substance metabolism [15,16,17]. However, to the extent of authors’ knowledge, little insights on relationship between “raw material–microorganism-VOCs” in the process of paocai fermentation are available, especially based on metagenomics sequencing technology.

Therefore, in this study, red radish and cabbage, the most common paocai raw materials in Sichuan, were used as raw materials for multiround fermentation, and the composition of microbial and volatile components in paocai fermentation systems were analyzed by metagenomics and gas chromatography–mass spectrometry (GC–MS), in order to reveal the effects of vegetable raw materials on the microbial community structure during Chinese homemade paocai multiple fermentation rounds and their relationship with volatile compounds.

## 2. Materials and Methods

### 2.1. Paocai Preparations

Red radish (*Red raphanus* L.) and cabbage (*Brassica oleracea* L.) were purchased from the local market in Ya’an, Sichuan Province, China. The fresh vegetables were cleaned under the same conditions to ensure the consistency of the initial flora on the surface of the vegetables. Paocai was made according to the local traditional method, specifically: one kilogram of each vegetable was cut into 2–3 cm pieces and set in 4 L pottery jars with garlic (3%), hot red peppers (3.5%), ginger (3%), and Chinese prickly ash (3%) (all percentages were calculated according to the volume of brine in the pottery jars, in *w*/*v*). Two liters of brine was prepared with cooled, boiled water containing 8% salt, 1% Chinese baijiu and 20% aged Sichuan paocai brine (in terms of *w*/*v*, collected from the homemade paocai of a local family used for more than 5 years). The pottery jars were sealed by adding water to the jar edge and stored at 24 ± 2 °C for six rounds of fermentation for about 9 d, 7 d, 5 d, 4 d and 3 d, respectively.

### 2.2. DNA Extraction, Library Construction and Metagenomic Sequencing

A total of 50 mL of initial aged brine and paocai brine at the end of each fermentation round were enriched on a 0.22 μm sterile microfiltration membrane. According to the manufacturer’s recommendation, the microbial DNA from brine samples was extracted with the E.Z.N.A.^®^ DNA Kit (Omega Bio-tek, Norcross, GA, USA). The concentration and purity of the DNA were determined with Turner Biosystems (Sunnyvale, CA, USA) and a NanoDrop2000 spectrophotometer (Thermo Fisher Scientific, TBS-380, Shanghai, China), and the integrity of the DNA was detected by 1% *w*/*w* agarose gel electrophoresis.

DNA was fragmented to an average size of about 400 bp using Covaris M220 (Gene Company Limited, Hong Kong, China) for paired-end library construction, and the paired-end library was constructed using NEXTflex^TM^ Rapid DNA-Seq (Bioo Scientific, Austin, TX, USA).

Adapters containing the full complement of sequencing primer hybridization sites were ligated to the blunt end of fragments. Paired-end sequencing was performed on Illumina NovaSeq (Illumina Inc., San Diego, CA, USA) at Majorbio Bio-Pharm Technology Co., Ltd. (Shanghai, China) using NovaSeq 6000 according to the manufacturer’s instructions. Sequence data associated with this project have been deposited in the NCBI Short Read Archive database (accession number: PRJNA731323).

### 2.3. Metagenome Data Integration

The raw reads from the metagenome sequencing were used to generate clean reads by removing adaptor sequences, trimming and removing low-quality reads (length < 50 bp, or with a quality value < 20 or having N-bases) using the fastp [18] (https://github.com/OpenGene/fastp, version 0.20.0, accessed on 1 December 2020) on the free online platform of Majorbio’s Cloud Platform (https://cloud.majorbio.com/, accessed on 1 December 2020). Megahit (parameters: kmer_min = 47, kmer_max = 97, step = 10) (http://www.l3-bioinfo.com/products/megahit.html, version 1.1.2, accessed on 6 December 2020) based on the principle of succinct de Bruijn graphs was used to assemble the optimized sequences [19]. Among the splicing results, contigs ≥ 300 bp were selected as the final assembly result. MetaGene was used to predict the open reading frames (ORFs) of contigs in the stitching results (http://metagene.cb.k.u-tokyo.ac.jp/, accessed on 11 December 2020) [20].

The predicted ORFs with lengths over 100 bp were retrieved and translated to amino acid sequences using the NCBI translation table (https://www.ncbi.nlm.nih.gov/Taxonomy/taxonomyhome.html/index.cgi?chapter=tgencodes#SG1, accessed on 15 December 2020). CD-HIT (http://www.bioinformatics.org/cd-hit/, version 4.6.1, accessed on 15 December 2020) was used to classify all sequences with 90% sequence identity and 90% coverage as nonredundant gene catalog [21]. Reads after quality control were mapped to the representative genes with 95% identity using SOAPaligner (https://github.com/ShujiaHuang/SOAPaligner, version 2.21, accessed on 20 December 2020) [22], and gene abundance in each sample was evaluated.

### 2.4. Species Annotation

Diamond (https://github.com/bbuchfink/diamond, version 0.8.35, accessed on 21 December 2020) was employed for taxonomic annotations by aligning nonredundant gene catalogs against the NCBI NR database using blastp as implemented in DIAMOND v0.9.19 with an e-value cutoff of 1 × 10^−5^ [23].

### 2.5. Determination of VOCs Using GC–MS

The analysis of the VOCs was performed by HS–SPME combined with GC–MS (7890A GC, Agilent, USA, 5975C MS; Agilent Technologies, Santa Clara, CA, USA). The paocai brine (5 mL) were accurately weighed into 15 mL headspace glass vials containing 1 g of salt, the vials were immediately placed in a heating block to equilibrate for 30 min at 50 °C and the VOCs were extracted using a 65 μm 50/30 um DVB/CAR/PDMS optical fiber for 10 min at 50 °C. VOCs were desorbed from the SPME fiber at 250 °C for 5 min in the injector in splitless mode. The carrier gas was helium, and it was supplied at a flow rate of 1.2 mL/min (constant linear velocity). The column temperature was programmed as follows: maintained at 36 °C for 3 min, increased at 5 °C/min to 65 °C, increased at 3 °C/min to 155 °C, increased at 10 °C/min to 200 °C, and maintained at 200 °C for 3 min. The MS condition was set as: 280 °C for the transfer line, 230 °C for the ionization, 70 eV for the ionization energy [10]. The quantification analysis was done by using cyclohexanone (27.424 g/L, *w*/*v*) as an internal standard. The NIST11.L standard mass spectral database was used to identify VOCs based on retention time and mass spectral similarity match. Each sample was analyzed in triplicates at each sampling time.

### 2.6. Statistical Analysis

The nonmetric multidimensional scaling analysis (NMDS), the analysis of similarities (ANOSIM), the heatmap and the bar plot were performed in the Vegan package of R (version 3.3.1). The differences between classes were analyzed using Linear discriminant analysis Effect Size (LEfSe) (http://huttenhower.sph.harvard.edu/galaxy/root?tool_id=lefse_upload, Version 1.0, accessed on 1 December 2020). SIMCA-P (Version14.0 (Umetrics, Umeå, Sweden) was used to analyze VOCs by partial least squares discriminant analysis (PLS-DA).

## 3. Results and Discussion

### 3.1. Metagenomic Sequencing Statistics and Quality Control

**Figure 1:**
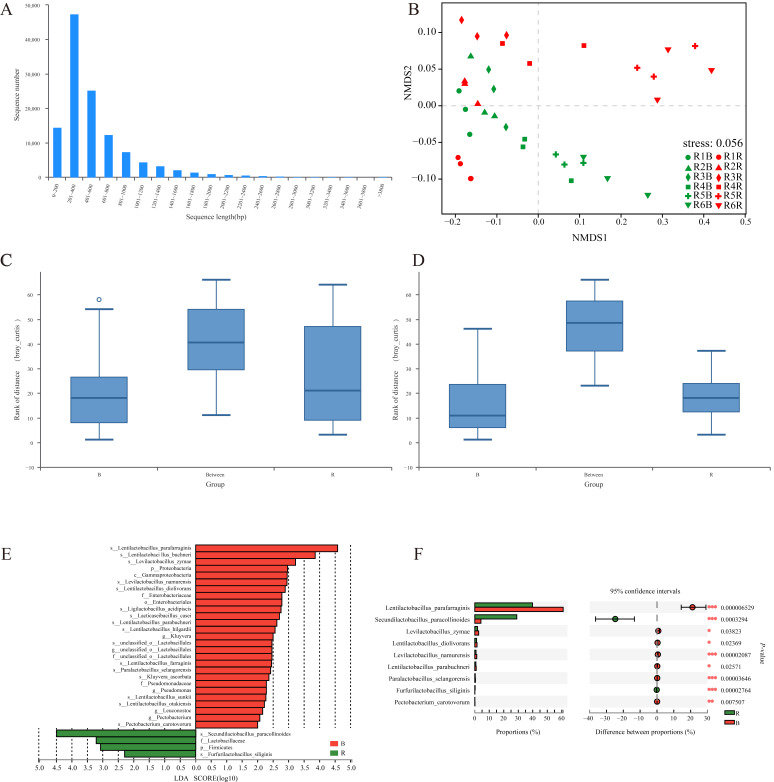
(**A**) Sequence length distribution of nonredundant gene catalog. (**B**) NMDS analysis at species level. Analysis of similarities between different groups by ANOSIM (**C**,**D**) of red radish paocai and cabbage paocai in the 3rd and 4th round, separately. (**E**) LDA score of the two kinds of red radish paocai and cabbage paocai. (**F**) Wilcoxon rank-sum test of different species in red radish paocai and cabbage paocai. * 0.01 < *p* ≤ 0.05, ** 0.001 < *p* ≤ 0.01, *** *p* ≤ 0.001.

A total of 39 samples were sequenced by the Illumina NovaSeq 6000 sequencing systems, and 1708.39 × 10^6^ raw reads, about 257.97 GB, with an average of 6.61 GB of each sample were generated. After the quality processing and eliminating the sample host gene, the sequence utilization of each sample was more than 98.48, including 787.85 × 10^6^ clean reads in the red radish samples and 776.19 × 10^6^ clean reads in the cabbage samples. A total of 966,355 contigs were assembled with Megahit, and the single sample’s contigs were between 9431 to 47,483. N50 and N90 were two indices for the distribution of contig lengths within a draft assembly, and the longer the assembly of samples the better. In all samples, the minimum value of N50 reached 1677 bp, the average value was 6680 bp, the N90 minimum was 421 bp, and the average value was 528 bp. Furthermore, a total of 1,778,731 genes were obtained by ORF prediction of the contigs, and the sequence length was 1,150,522,526 bp (Table S1). All predicted genes were clustered using CD-HIT (parameters: identity, 95; coverage, 90), resulting in a total of 122,012 nonredundant gene set. Figure 1A shows the sequence length distribution of nonredundant gene sets, mostly concentrated between 201~600 bp, with an average sequence length of 557.88 bp, and the information was used for subsequent species and functional annotations to reveal microbial community of samples.

### 3.2. Microbial Structure

The sequences comprising the total reads corresponded to five domains, eight kingdoms, 47 phyla, 99 classes, 207 orders, 353 families, 706 genera, and 2068 species, of which four domains were known. These four were identified to be bacterial, fungal, archaeal and virus domains, of which the bacterial domain had the highest proportion at 99.31% and the lowest was for the fungal domain. In addition, eight kingdoms in all samples were detected, of which the major phyla were *Firmicutes* and *Ascomycota* with *Firmicutes* the most abundant, contributing to over 98% in all samples, consistent with previous reports of other fermentation vegetables [24,25]. Further, it was interesting to note that with the progress of fermentation, the content of *Ascomycota* decreased gradually in the red radish samples, but there was no obvious change in the cabbage samples. The major classes, order and family were *Bacilli* and *Saccharomycetes*, *Lactobacillales* and *Saccharomycetales* and *Lactobacillaceae* and *Saccharomycetaceae*, consistent with the phyla identified.

**Figure 2:**
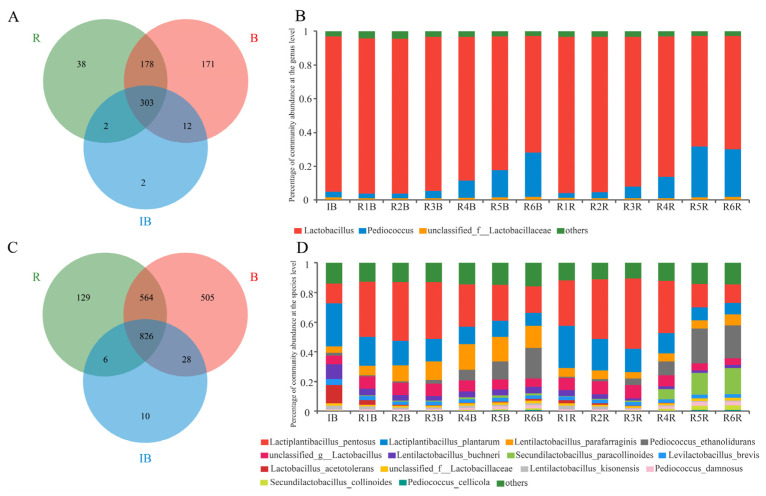
Venn plot (**A**) and bar plot of relative abundance (**B**) of microbial communities at the species level in the initial brine and two kinds of paocai and (**C**,**D**) at the genus level in the initial brine and two kinds of paocai. R indicates red radish paocai, B indicates cabbage paocai, IB indicates initial brine. Note: others in B and D means other microbes.

As for the genus level, a total of 706 microbe genera were detected, of which 319, 521 and 664 genera were detected in the initial red radish and cabbage brines, respectively. Among them, 303 genera occurred in all paocai samples, wherein 38 genera only appeared in the red radish samples, 171 genera only appeared in the cabbage samples and 303 genera appeared in both paocai samples (Figure 2A). The microbial diversity of paocai was found to be relatively simple, and there were only three genera with abundance more than 1% of the diversity, and they belonged to the *Lactobacillus*, *Pediococcus* and an unclassified *Lactobacillaceae* genus. The average abundance of *Lactobacillus* in the initial brine, red radish paocai and cabbage paocai reached 92.35%, 85.10% and 81.84%, respectively. Furthermore, *Lactobacillus* was the predominant bacteria in the multiround fermentation, and was found to be in agreement with other paocai studies [8]. However, with the progress of fermentation, *Lactobacillus* gradually decreased, accounting for only 67.4% of red radish paocai at the end of the sixth round of fermentation and 69.5% of cabbage paocai. Although previous studies have different conclusions about whether *pediococcus* is the dominant bacterium in fermented vegetables [12,26,27], the proportion of *Pediococcus* genus was found in general to be increasing along with the fermentation. Specifically, its abundance was found to increase from 2.82% to 28.37% in the red radish and 2.58% to 26.28% in the cabbage paocai brines (Figure 2B), respectively. These data indicate that although they belong to the same phylum (Firmicutes), *Pediococcus* may be more stress-resistant or competitive than *Lactobacillus* in the later stage of fermentation.

At the species level, a total of 2068 microbial species were detected by sequencing, of which 870, 1525 and 1923 species were detected in the initial, red radish and cabbage brines. Among the detected species 826 of them occurred in all paocai samples (Figure 2C), whereas 129 species appeared only in the red radish samples, and 505 species only in the cabbage samples. A further 564 species commonly appeared in all the red radish and cabbage paocai samples, but only 11 of them were recorded with relative abundances of more than 1%. In addition, a higher number of microbial species were present in the cabbage paocai than in the red radish paocai with a brine of the same age; moreover, with the increase of fermentation time, the number of microorganisms in the red radish paocai increased first and then decreased, while the number of microorganisms in the cabbage paocai did not obviously change, but after six rounds of fermentation, the number of microorganisms in the cabbage paocai was significantly higher than that in the red radish paocai.

As for the individual detected species, the *Lentilactobacillus buchneri* (9.9%) and *Lactobacillus acetotolerans* (12.5%) in the initial brine were found to be the dominant species. *Lactiplantibacillus paraplantarum* species were found to be gradually decreasing with the progression of fermentation rounds from 29.1% to 8.6% in the cabbage paocai and from 29.1% to 7.9% in the red radish paocai samples. With regard to the *Pediococcus ethanlidurans*, *Pediococcus damnosus* and *Secundilactobacillus paracollinoides* species, their proportions were found to be gradually increasing with fermentation rounds. Specifically, *P. ethanlidurans* species registered a significant increase from the first to the last round of fermentation from 0.7% to 20.67% in the red radish and 0.94% to 22.39% in the cabbage paocai samples, respectively. This occurrence indicates that *P. ethanlidurans* might have a better growth competitiveness compared to *L. paraplantarum* under the final round of fermentation conditions characterized by a high salinity and acidity.

Further, *Lactiplantibacillus pentosus,* which is used as the starter in the production of homemade paocai to improve the quality [5], was observed to be the dominant strain, and increased gradually until the third round of fermentation, then decreased continuously until the last round of paocai fermentation (from 38.8% to 18.0% and from 47.4% to 12.2% in red radish samples and cabbage samples, respectively). This could be due to the preparation process and some limitations as reported in previous studies [28,29]. The microbial community in the later stages of fermentation for both the red radish paocai and cabbage paocai was found to be similar. The proportion of *Lentilactobacillus parafarraginis* was found to be relatively higher in the cabbage paocai (15.2%) than that in the red radish paocai (7.3%), whereas the relative abundance of *S. paracollinoides* was higher in the red radish paocai (17.7%) than that in the cabbage paocai (1.7%) (Figure 2D).

### 3.3. Microbial Composition Comparison

**Figure 3:**
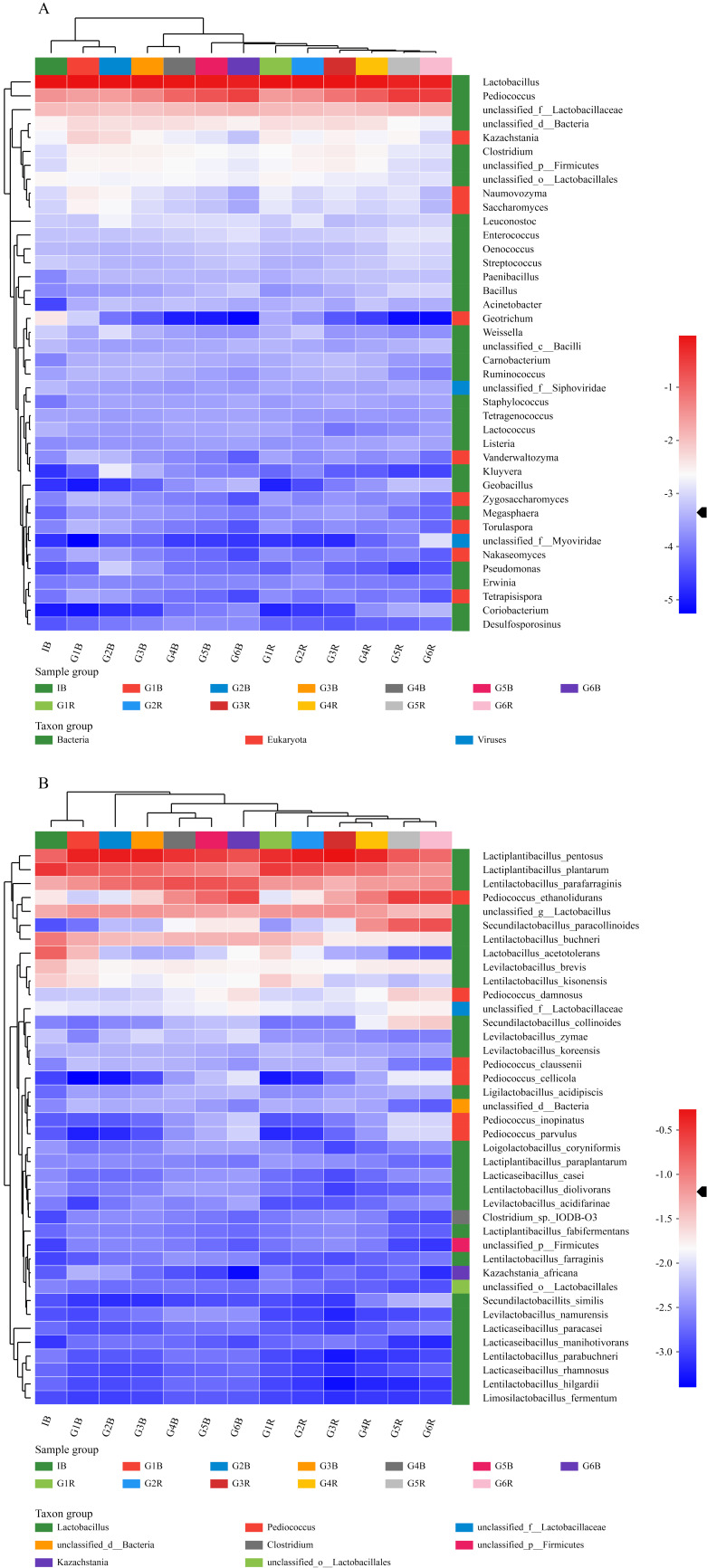
(**A**) The heatmap analysis of the top 40 microorganisms at the genus level. (**B**) The heatmap analysis of the top 40 microorganisms at the species level.

The heatmap analysis was utilized to reveal the relative relationship of the top 40 microbial genera detected with the colors representing the species abundance (Figure 3A). It can be clearly observed from the heatmaps that in both evaluated samples, the abundance of *Lactobacillus* and *Pediococcus* in bacteria and *Kazachstania* in eukaryotes was relatively stable during all six rounds of fermentation. Moreover, *Weissella* was found to be relatively rich during the whole fermentation process, but its relative abundance decreased as the fermentation progressed. This decrease in *Weissella*’s abundance can be attributed to the increase in the types of species and the subsequent competition for nutrients, as was also observed in other studies [30,31]. In addition, the abundance of *Geotrichum* and *Kazachstania* decreased gradually during the fermentation in the two fermented vegetables, whereas the abundance of *Enterococcus*, *Streptococcus*, *Clostridium* and *Bacillus* showed a trend toward a gradual increase in the fermentation process, with no change in their relative abundance in both fermentation systems even though they exhibited significantly opposite microorganisms.

In fact, metagenomics sequencing allows the obtained data to be compared with a database to extract the information of microorganisms at the species level [32,33]. Within the top 40 abundant species detected (Figure 3B), 30 species belonged to the genus of LAB. Within the LAB species, a varying behavior of abundance was observed along the fermentation process with species such as *L. paraplantarum*, *Levilactobacillus brevis*, *L. acetotolerans* decreasing in abundance during the fermentation in both samples.

A UPGMA cluster analysis of the samples was performed based on the identified genus and species, demonstrating the higher similarity between the red radish samples.

To further understand the microbiota structure during paocai fermentation, NMDS and LDA Effect Size (LEfSe) was used to analyze the microbial differences. The NMDS analysis (Figure 1B) showed that the microbial community of the red radish and cabbage paocais displayed an obvious spatial structure during the fermentation process, where the composition of microorganisms in different vegetable fermentation systems exhibited greater differences (stress = 0.016 < 0.05). With the progress of the fermentation, the distance between spatial positioning of different fermented vegetable samples of the same round gradually increased, indicating that the influence of raw materials on the microbial composition increased with the increase of fermentation time. These observed results were consistent with the ANOSIM analysis, with the R value closer to 1 confirming the greater differences between groups with a *p*-value less than 0.05 indicative of the high reliability of the test (Figure 1C,D). Towards the end of the third and fourth rounds of paocai fermentation, the different samples were significantly separated (R = 0.46, *p* = 0.009) with a further increase in distance at the end of the fifth and the sixth fermentation rounds (R = 0.89, *p* = 0.003). The aforementioned data further confirm that the microbial composition of different vegetable paocais presented continuous changes in the multiround fermentation with the differences significantly increasing along the fermentation time.

LEfSe (Figure 1E) was used as an algorithm for high-dimensional biomarker discovery and identifying genomic features characterizing the differences between two or more biological conditions [34]. In this study, LEfSe was performed to evaluate the differences between the bacterial community structures of the different paocais. In the paocai, a total of 31 taxa were found to represent a remarkable difference in their relative abundance, with an LDA (log10) > 2. At the species level, *S. paracollinoides* and *Furfurilactobacillus siliginis* were found to be the characteristic bacteria of the red radish paocai, whereas a total of 15 species of microbes were found characteristic of the cabbage paocai, of which 13 species belonged to the Firmicutes (namely, *L. parafarraginis*, *L. buchneri*, *Levilactobacillus zymae*, *Levilactobacillus namurensis*, *Lentilactobacillus diolivorans*, *Ligilactobacillus acidipiscis*, *Lacticaseibacillus casei*, *Lentilactobacillus parabuchneri*, *Lentilactobacillus hilgardii*, *Lentilactobacillus farraginis*, *Paralactobacillus selangorensis*, *Lentilactobacillus sunkii*, *Lentilactobacillus otakiensis*) and two species to that of Proteobacteria (namely, *Kluyvera ascorbata*, *Pectobacterium carotovorum*). Further, the differential microorganisms were screened by a Wilcoxon ranksum test (Figure 1F), which revealed that only nine species were significantly different in the red radish paocai and cabbage paocai, with *L. parafarraginis*, *S. paracollinoides*, *L. namurensis*, *P. selangorensis* and *F. siliginis* being the significantly different species in the paocais tested. In fact, all the nine species detected were also screened by LEfSe and regarded as biomarkers for the subsequent analysis.

### 3.4. Profiles of VOCs during Different Paocai Fermentations

**Figure 4:**
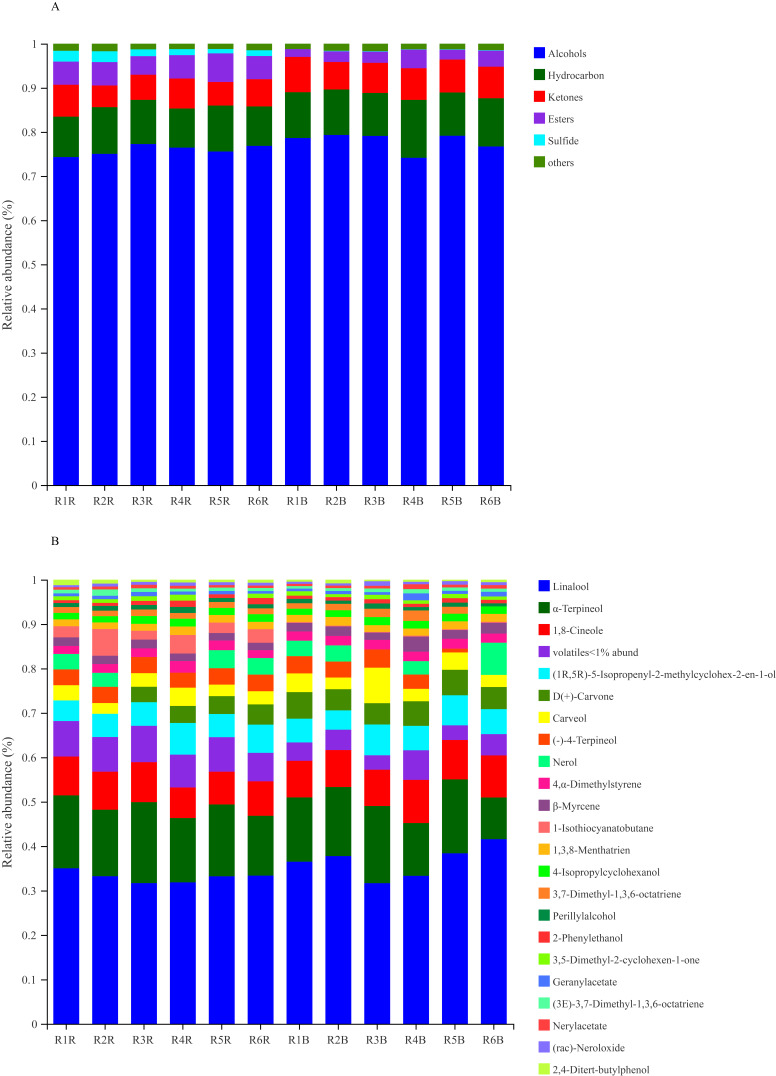
(**A**) Changes of VOCs in red radish paocai and cabbage paocai during fermentation. (**B**) Major VOCs with relative abundance of more than 1% of the red radish paocai and cabbage paocai.

The compositions of VOCs in the paocai samples are shown in Table S2. A total of 62 and 59 volatile substances were identified in the red radish and cabbage paocais. As shown in Figure 4A, the main VOCs detected were alcohols, hydrocarbons, esters, sulfide and ketones during the whole fermentation process. Alcohols were the most abundant compounds among those detected and ranged from 74.1% to 79.3%, with a total of 17 different components. The second largest group was heterocycles which ranged from 9.8% to 13.1% and included 23 component types.

Figure 4B shows 22 major VOCs that accounted for more than one of the two paocai volatile profiles. In general, the types of VOCs identified were similar, both types included compounds like linalool, α-terpineol, eucalyptol, d-carvone and other substances. Among these, linalool registered the highest proportions reaching up to 35% and 41.6% in the red radish and cabbage paocais, respectively, similar to previous studies [8]. Linalool contributes to the unique flavor of paocai and is described as having a strong scent, similar to bergamot oil or French lavender, with a mixture of woody, floral aromas with a touch of spiciness [35]. Other alcohols were also evaluated to have a positive effect on paocai’s flavor with α-terpineol contributing a floral typically lilac odor; eucalyptol with a eucalyptus, herbal and camphor odor; and nerol with notes of roses [36,37].

Red radish and cabbage belong to the Cruciferae family of plants, and typically have a strong aroma, bitter taste and pungent taste similar to that of mustard. The compounds responsible for these special flavors were found to be mainly the secondary defense metabolite, glucosinolate and its hydrolysate isothiocyanate [38]. The composition and content of isothiocyanates is variable based on the type of vegetables subsequently imparting characteristic flavor components and compositions in the fermented paocais. Both tested paocais were found to contain butyl isothiocyanate and amyl isothiocyanate, with significantly higher values in the red radish paocai. Among the sulfides, dimethyl disulfide and dimethyl trisulfide were uniquely found in the red radish paocai, whereas 3-butenylisothiocyanate was found to be the unique sulfide in the cabbage paocai.

Among the various potential detected substances, those with a low content (lower than the threshold values) were excluded in the statistical analysis to narrow down the range of key volatiles. In detail, 36 potential volatile substances were screened out, which included 14 alcohols, 3 ketones, 5 esters, 10 hydrocarbons, 3 sulfides, and 1 phenol compounds.

**Figure 5:**
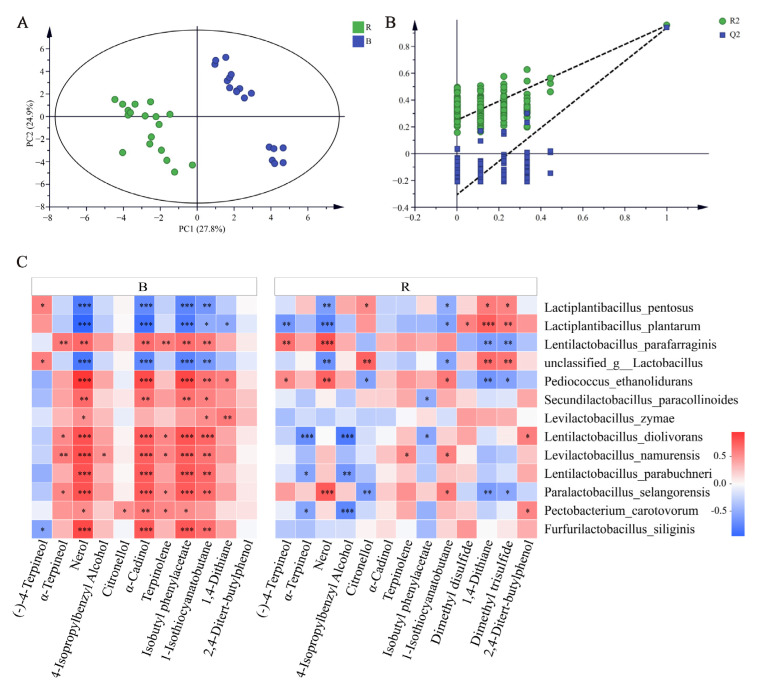
(**A**) PLS-DA score scatter plot and (**B**) permutation tests of VOCs. (**C**) Heatmap of correlation between microbial species (relative abundance exceeding 5% and the nine biomarkers previously identified) and different volatile flavor substances in the two kinds of paocai. R indicates red radish paocai, B indicates cabbage paocai. * 0.01 < *p* ≤ 0.05, ** 0.001 < *p* ≤ 0.01, *** *p* ≤ 0.001.

To further screen the different VOCs in two kinds of paocai, a PLS-DA model was established, as shown in Figure 5A,B. Variable projection importance (VIP) was used to evaluate the contribution of variables. Substances with VIP value ≥1.0 were selected and combined with the conditions with a significant difference between the two groups (*p* < 0.05). The point distance between the VOCs of paocai fermented with two different raw materials was obvious, which indicated that the VOCs of paocai fermented with different raw materials were different. The total contribution rate of the first two principal components on variable X was 52.7% (R_2_X = 0.527), and the contribution rate on variable Y was 95.7% (R_2_Y = 0.957). At the same time, the prediction ability based on cross-validation was 94.0% (Q_2_ = 0.94), indicating that the model had a good explanatory degree. The stability and reliability of this model were verified by the cross-validation model with 200 permutation validations. As shown in Figure 5B, all Q_2_ values on the left were lower than the original point on the right, and the regression line of point Q_2_ intersected with the vertical axis below zero, and the intercept of −0.306 was less than 0.05, indicating that this PLS-DA model did not exhibit over-fitting and had good representativeness. A total of 13 compounds were screened out as the difference substances and included compounds like (−)-4-terpineol, α-terpineol, nerol, 4-isopropylbenzyl alcohol, carveol, α-cadinol, terpinolene, isobutyl phenylacetate, 1-isothiocyanatobutane, dimethyl disulfide, 1,4-dithiane, dimethyl trisulfide and 2,4-ditert-butylphenol.

Further to explore the correlation between microorganisms and VOCs, a Spearman correlation analysis was carried out with a relative abundance exceeding 5% and the nine biomarkers previously identified. It can be seen from Figure 5C that the microorganisms have different effects on the VOCs of paocai fermented by two kinds of vegetables. Nerol was also significantly correlated with six kinds of microorganisms in the red radish paocai to different degrees. In the cabbage paocai, the correlations between nerol, α-cadinol, isobutyl phenylacetate and the microorganisms were consistent, that is, they have a significant positive correlation with seven species and a significant negative correlation with three species at the same time, indicating that these 13 microorganisms have the same regulatory effect on the changes of these three substances. In addition, 1-isothiocyanatobutane, 1,4-dithiane and dimethyl trisulfide, which account for a high proportion of compounds in the red radish paocai, were related to six kinds of microorganisms, especially *L. paraplantarum* species. However, 2,4-ditert-butylphenol in the cabbage paocai and α-cadinol in the red radish paocai were not related to microorganisms.

From the perspective of the relationship between microorganisms and VOCs, *F. siliginis*, *L. parafarraginis*, *L. namurensis*, *L. zymae* and *S. paracollinoides* were found to be the key microorganisms for distinguishing the two kinds of paocai, and 4-isopropylbenzyl alcohol, α-cadinol, terpinolene and isobutyl phenylacetate were the key VOCs for distinguishing the two kinds of paocai made from red radish and cabbage.

## 4. Conclusions

In this paper, metagenomic sequencing and a HS–SPME method based on a GC–MS method were used to systematically study the changes of microbial flora and VOCs in paocai fermented with different vegetables in six rounds of fermentation, combined with a multivariate statistical method to explore the intrinsic associations of “raw materials—microorganism—VOCs”. The results revealed that after multiple rounds of fermentation, a higher number of microbial species were present in the cabbage paocai than in the red radish paocai with a brine of the same age. At the species level, *S. paracollinoides* and *F. siliginis* were found to be the characteristic microbial species of the red radish paocai, as well as 13 species belonging to the Firmicutes phylum (namely, *L. parafarraginis*, *L. buchneri*, *L. zymae*, *L. namurensis*, *L. diolivorans*, *L. acidipiscis*, *L. casei*, *L. parabuchneri*, *L. hilgardii*, *L. farraginis*, *P. selangorensis*, *L. sunkii* and *L. otakiensis*) and two species belonging to Proteobacteria (namely, *K. ascorbata* and *P. carotovorum*) for the cabbage paocai. By constructing a PLS-DA model, it was found that there were 13 kinds of different VOCs in the paocai from two raw materials. In addition, the correlation between the microorganisms and VOCs in the cabbage paocai was stronger than that in the red radish paocai, and the most significant differences were found to be in 4-isopropylbenzyl alcohol, α-cadinol, terpinolene and isobutyl phenylacetate. The metagenomic sequencing in this study allowed the detection, identification and screening of the microbial ecology as well as the characteristic microorganisms of two different fermentation systems of the vegetable paocais. Further, the study also effectively allowed the identification of the VOCs and the establishment of their relationship to the microorganisms, which provided insights into the microbial flavor profiles of the studied paocais.

## Data Availability

The data presented in this study are openly available in NCBI database at accession PRJNA731323. deposited in https://www.ncbi.nlm.nih.gov/sra/PRJNA731323 (accessed on 21 December 2020).

## Supplementary Materials

The following are available online at https://www.mdpi.com/article/10.3390/foods11010062/s1, Table S1: Statistics profiles of the metagenome, Table S2: Content of volatile organic compounds in the two kinds of paocai.
