# Supplementary material for: Metagenomic Study on Chinese Homemade Paocai: The Effects of Raw Materials and Fermentation Periods on the Microbial Ecology and Volatile Components
Source: Foods. 2021 Dec 28;11(1):62. doi: 10.3390/foods11010062 (PMC8750508; doi:10.3390/foods11010062)
Supplement: Supplementary file 1 [file foods-11-00062-s001.zip › foods-1473488-supplementary.pdf]

Table S1. Statistics profiles of the metagenome. Note: Raw Reads: the length of raw reads; Clean Reads: the filtered sequence length; Cleaned (%): the content of filtered sequence number; Clean Base represnet the number of sequence base; Contigs: sequence of contigs; N50(N90): each contigs sequence is sorted by length, and the length value of each sequence is accumulated one by one from large to small. When the cumulative value first exceeds 50% (90%) of the total length of all sequences, the length value of the sequence is scanned.
Table S2. Content of volatile organic compounds in the two kinds of paocai.

Table S1. Statistics profiles of the metagenome.

Sample	Raw Reads ($\times 10^6$)	Clean Reads ($\times 10^6$)	Cleaned(%)	Clean Base ($\times 10^9$ bp)	Contigs	N50 (bp)	N90 (bp)
IB1	42.26	41.91	99.16	6.32	27138	9157	423
IB2	42.03	41.69	99.19	6.28	25212	9960	426
IB3	44.59	44.25	99.24	6.67	34511	5611	421
R1B1	46.65	46.22	99.08	6.97	36039	2605	542
R1B2	43.06	42.69	99.13	6.44	36173	2659	531
R1B3	43.00	42.57	99.01	6.42	31213	2798	486
R1R1	40.53	40.11	98.98	6.05	26233	3678	485
R1R2	40.43	40.04	99.03	6.04	32616	2082	473
R1R3	48.26	47.85	99.16	7.22	33814	2382	500
R2B1	42.70	42.36	99.19	6.39	47483	1677	432
R2B2	51.95	51.33	98.81	7.74	38390	2402	461
R2B3	43.26	42.80	98.95	6.46	37967	2963	502
R2R1	45.90	45.20	98.48	6.82	21492	5828	484
R2R2	42.92	42.48	98.96	6.41	23909	7873	485
R2R3	43.43	43.00	99.00	6.49	30817	2941	433
R3B1	43.08	42.69	99.09	6.44	26594	4308	461
R3B2	43.34	42.95	99.10	6.48	30312	2528	483
R3B3	42.83	42.34	98.86	6.39	17566	10410	514
R3R1	40.25	39.83	98.96	6.01	23925	6445	497
R3R2	43.25	42.77	98.87	6.45	23915	6899	506
R3R3	42.75	42.31	98.96	6.38	29600	1917	471
R4B1	42.94	42.58	99.17	6.42	16191	12455	522
R4B2	53.02	52.56	99.13	7.92	27864	3302	471
R4B3	46.99	46.61	99.19	7.03	18993	11674	508
R4R1	42.95	42.50	98.95	6.41	24220	5753	486
R4R2	42.74	42.35	99.08	6.39	17785	10812	535
R4R3	42.40	42.04	99.15	6.34	22124	5152	491
R5B1	40.20	39.91	99.27	6.02	14612	12652	573
R5B2	42.61	42.17	98.96	6.36	18612	6290	517
R5B3	42.27	41.92	99.18	6.32	12479	15405	656
R5R1	41.41	41.04	99.11	6.19	30022	2394	486
R5R2	41.37	41.02	99.16	6.19	22114	8563	505
R5R3	43.16	42.76	99.08	6.45	27698	2554	468
R6B1	44.58	44.25	99.25	6.67	10599	13576	842
R6B2	42.00	41.54	98.91	6.26	9431	16491	1013
R6B3	40.78	40.36	98.98	6.08	11751	9507	782
R6R1	47.13	46.48	98.63	7.01	14116	9279	596
R6R2	54.45	53.96	99.10	8.14	14774	12432	588
R6R3	40.91	40.45	98.87	6.10	18051	5113	519

Total	1708.39	1691.89	99.03	255.14	966355	6680	528

Table S2. Content of volatile organic compounds in the two kinds of paocai. Volatile compounds analyzed by HS-SPME/GC–MS during paocai fermentation.

Volatile Compounds	CAS Number	RT (min)	Concentration (mg/kg)											
			R1R	R2R	R3R	R4R	R5R	R6R	R1B	R2B	R3B	R4B	R5B	R6B
Alcohols														
Carveol	000099-48-9	13.84	55.55	48.09	67.82	83.93	61.84	64.60	60.58	36.78	89.63	65.83	78.24	55.54
Eucalyptol	000470-82-6	14.73	143.99	170.10	199.45	140.67	176.45	169.52	118.73	116.38	194.10	212.36	177.50	192.50
Linalool	000078-70-6	17.47	602.84	659.73	701.66	738.00	750.57	727.98	501.60	528.80	751.11	784.18	769.56	746.12
2-Phenylethanol	000060-12-8	17.74	11.07	13.67	20.43	28.93	19.36	30.80	10.35	10.19	22.81	17.52	20.23	15.92
4-Isopropylcyclohexanol	004621-04-9	18.34	22.58	28.24	39.13	35.74	43.53	37.64	20.17	21.36	43.45	40.53	34.43	34.52
β-Terpineol	000138-87-4	18.57	7.94	10.89	8.57	6.88	10.20	7.72	5.87	5.19	7.38	8.77	6.50	6.22
(-)-4-Terpineol	020126-76-5	19.53	58.06	72.61	80.77	69.02	88.47	81.83	46.63	50.48	97.72	75.80	17.13	1.80
α-Terpineol	000098-55-5	20.05	282.43	297.27	403.71	344.47	386.91	293.70	208.45	218.10	292.03	279.25	309.44	229.53
(Z)-Carveol	001197-06-4	20.86	80.11	103.78	117.92	145.59	136.31	138.65	76.90	77.63	164.34	129.42	135.76	114.54
Nerol	000106-25-2	21.23	59.78	62.61	59.76	79.76	97.15	81.22	10.74	14.57	20.82	37.01	42.41	90.97
4-Isopropylbenzyl Alcohol	000536-60-7	22.81	7.17	10.12	13.77	14.49	12.02	10.01	6.26	4.55	11.04	8.62	9.02	7.21
Perillyl alcohol	000536-59-4	23.00	15.64	21.29	22.62	28.01	24.97	20.29	14.27	10.32	28.34	18.12	18.60	14.08
Nerolidol	040716-66-3	31.08	0.27	0.43	0.33	0.16	0.16	-	0.16	0.12	0.14	0.16	-	0.17
Citronellol	000106-22-9	31.44	3.51	4.18	4.41	3.19	3.43	2.74	1.35	1.86	0.89	2.26	1.43	1.35
Spathulenol	006750-60-3	31.61	-	-	8.39	8.59	6.89	-	-	3.23	-	5.28	4.44	5.13
γ-Eudesmol	001209-71-8	32.99	0.93	1.33	1.30	1.34	1.14	1.39	-	0.56	-	1.03	-	1.04
α-Cadinol	000481-34-5	33.79	3.44	3.79	3.69	3.57	4.77	3.75	1.43	1.77	1.82	2.83	2.61	3.35
Ketones														
D(+)-Carvone	002244-16-8	21.48	111.24	59.87	76.93	117.41	96.46	99.59	86.23	66.88	114.09	123.80	115.33	101.50
3,5-Dimethyl-2-cyclohexen-1-one	001123-09-7	22.24	15.13	16.37	23.21	27.37	25.46	21.26	14.31	9.35	23.57	18.23	19.15	15.40
2,6,6-Trimethylcyclohepta-2,4-dien-1-one	000503-93-5	24.20	5.51	8.64	9.45	9.23	6.94	-	4.40	4.25	-	8.29	8.97	7.25

Table S2 (continued)

**Volatile Compounds**	**CAS Number**	**RT (min)**	**Concentration (mg/kg)**											
			**R1R**	R2R	R3R	R4R	**R1R**	R6R	R1B	R2B	**R1R**	R4B	R5B	R6B
Jasmolin I	004466-14-2	32.03	-	13.83	19.65	-	-	13.46	5.38	6.05	11.35	13.08	10.10	17.65
Hydrocarbon														
β-Myrcene	000123-35-3	13.43	33.42	37.44	43.81	34.53	40.20	36.58	27.65	29.96	38.86	80.07	39.68	49.39
α-Terpinene	000099-86-5	14.24	-	4.01	4.08	-	-	3.63	3.29	3.74	-	-	3.70	4.07
4-Carene	029050-33-7	14.25	11.03	-	-	-	4.29	-	3.20	3.20	4.58	-	-	-
(3E)-3,7-Dimethyl-1,3,6-octatriene	003779-61-1	15.02	13.95	28.30	18.13	13.06	18.07	15.08	8.10	8.88	19.32	22.40	15.13	14.58
3,7-Dimethyl-1,3,6-octatriene	013877-91-3	15.35	20.49	24.39	32.40	26.23	31.67	28.70	17.71	20.18	44.49	55.25	31.45	10.16
γ-Terpinene	000099-85-4	15.68	1.80	1.94	2.19	1.92	2.72	1.83	1.55	1.96	2.39	3.43	2.17	2.28
(4E,6Z)-2,6-Dimethylocta-2,4,6-triene	007216-56-0	18.01	8.47	9.31	10.81	8.73	11.27	8.28	7.06	6.93	-	22.86	-	9.11
Terpinolene	000586-62-9	19.11	4.40	5.87	5.72	5.30	7.89	7.24	2.94	3.14	4.22	6.13	4.20	3.11
7-Methylidene-1,2,3,3a,4,5,6,7a-octahydroindene	040954-37-8	22.63	0.09	0.17	0.14	-	-	-	-	-	-	0.34	-	-
a-Bornene	000464-17-5	24.41	-	10.36	-	-	-	-	5.86	-	-	-	10.10	17.40
5-methyl-3-prop-1-enylcyclohexene	056816-08-1	26.23	-	7.37	8.39	-	10.14	-	4.35	3.15	2.06	2.82	4.29	3.46
(−)-allo-Aromadendrene	025246-27-9	28.07	0.49	0.86	0.99	0.48	0.76	0.96	0.83	0.35	0.21	1.31	0.26	0.42
Cenicriviroc sulfone	000495-60-3	29.08	2.69	1.78	4.25	4.91	6.08	3.34	1.27	1.44	2.40	3.27	3.83	5.43
(S)-β-Bisabolene	000495-61-4	29.42	1.67	1.59	1.96	1.64	1.73	1.36	1.04	0.54	1.06	4.17	1.13	1.34
δ-Cadinene	000483-76-1	29.89	3.42	3.97	6.51	3.83	6.69	3.37	2.18	1.35	2.42	9.60	2.57	3.47
1,3,8-Menthatrien	018368-95-1	14.09	26.65	29.40	34.33	39.15	43.91	35.98	23.45	27.30	35.91	37.68	35.63	36.35
4,α-Dimethylstyrene	001195-32-0	16.71	30.98	39.20	43.13	53.61	54.75	38.90	30.05	29.98	50.96	41.40	44.60	51.21
2-Methyl-trans-3-hexene	000692-24-0	27.73	2.20	2.42	5.34	-	2.32	2.04	-	-	-	-	-	-
α-Curcumene	000644-30-4	28.73	3.48	-	3.13	2.66	5.90	2.66	1.29	0.00	1.95	6.10	1.82	2.65

Table S2 (continued)

Volatile Compounds	CAS Number	RT (min)	Concentration (mg/kg)											
			**R1R**	R2R	R3R	R4R	**R1R**	R6R	R1B	R2B	**R1R**	R4B	R5B	R6B
α-Calacorene	021391-99-1	30.49	0.57	0.55	1.37	2.49	2.27	1.03	0.58	0.54	1.16	1.50	1.04	1.10
1,6-Dimethyl-4-propan-2-ylnaphthalene	000483-78-3	34.34	0.33	0.31	0.63	0.33	0.54	0.18	0.10	0.19	0.39	0.23	0.21	0.26
Guaiazulene	000489-84-9	36.89	0.14	0.19	0.33	0.36	0.10	0.15	-	-	0.14	0.07	0.08	-
γ-Muurolene	030021-74-0	33.54	1.01	3.42	-	1.82	0.45	3.31	0.68	0.52	-	0.75	-	0.98
Esters														
Ethyl butyrate	000105-54-4	6.58	2.46	2.90	1.27	1.24	1.14	-	0.99	0.42	0.46	-	-	-
4-Isothiocyanatobut-1-ene	003386-97-8	13.16	0.00	0.96	-	-	-	-	-	-	-	-	2.34	4.00
3-(Methylthio)Propyl Isothiocyanate	000505-79-3	23.31	3.82	10.70	2.96	6.75	6.05	6.04	3.04	2.80	7.43	9.42	9.66	10.89
Terpinyl acetate	000080-26-2	24.41	15.71	-	-	12.63	23.79	12.15	-	8.37	12.29	24.42	-	-
Neryl acetate	000141-12-8	24.86	9.44	13.07	17.64	13.22	13.27	12.02	7.36	8.10	14.28	16.28	12.70	25.48
Geranyl acetate	000105-87-3	25.52	14.24	15.42	21.45	14.59	24.02	12.75	8.52	10.15	14.27	36.90	14.06	21.51
Isobutyl phenylacetate	000102-13-6	25.82	2.43	3.42	5.21	3.44	4.20	3.33	1.39	1.39	2.32	3.06	2.42	3.17
1-Isothiocyanatobutane	000592-82-5	27.26	43.16	59.38	44.22	66.34	75.59	66.59	2.42	2.45	3.67	4.96	3.54	4.49
1-Pentylisothiocyanate	000629-12-9	30.60	1.61	0.45	2.26	1.43	7.34	1.47	0.41	0.05	0.49	0.96	0.58	1.29
Ethyl myristate	000124-06-1	37.17	2.76	0.09	0.26	0.16	0.66	-	0.13	-	0.09	-	-	0.37
9-Heyadecenoic acid ethyl ester	054546-22-4	41.08	-	0.07	0.12	0.20	-	-	-	-	-	-	0.21	0.70
Ethyl hexadecanoate	000628-97-7	41.50	0.15	0.23	0.19	0.11	0.53	0.04	0.06	0.05	0.09	0.22	0.32	0.43
Sulfide														
Dimethyl disulfide	000624-92-0	4.63	24.97	25.89	15.48	20.55	11.84	19.83	-	-	-	-	-	-
1,4-Dithiane	000505-29-3	10.68	9.24	10.66	8.41	3.64	5.12	0.00	-	1.57	2.23	1.60	1.85	2.01
Dimethyl trisulfide	003658-80-8	12.55	10.37	12.62	11.03	6.57	6.59	8.46	-	-	-	-	-	-
Others														

Table S2 (continued)

**Volatile Compounds**	**CAS Number**	**RT (min)**	**Concentration (mg/kg)**											
			**R1R**	R2R	R3R	R4R	**R1R**	R6R	R1B	R2B	**R1R**	R4B	R5B	R6B
Neroloxide	001786-08-9	18.76	8.02	13.16	14.50	15.33	15.79	13.98	6.86	7.02	24.43	13.02	15.98	13.44
2,4-Ditert-butylphenol	000096-76-4	29.67	21.42	17.48	10.96	12.80	13.69	14.88	6.66	11.62	8.45	11.24	6.57	11.47
(-)-Caryophyllene oxide	001139-30-6	32.14	-	3.99	3.82	-	-	3.63	3.43	3.74	4.20	4.66	3.70	4.07
